# Upregulated bone morphogenetic protein 8A (BMP8A) in triple negative breast cancer (TNBC) and its involvement in the bone metastasis

**DOI:** 10.3389/fcell.2024.1374269

**Published:** 2024-07-19

**Authors:** Laijian Sui, Yizi Cong, Ming Liu, Xiangyi Liu, Yali Xu, Wen G. Jiang, Lin Ye

**Affiliations:** ^1^ Cardiff China Medical Research Collaborative, Division of Cancer and Genetics, Cardiff University School of Medicine, Cardiff, United Kingdom; ^2^ Department of Orthopedics, Yantai Yuhuangding Hospital, Yantai, Shandong, China; ^3^ Department of Breast Surgery, Yantai Yuhuangding Hospital, Qingdao University, Yantai, China

**Keywords:** BMP8A, breast cancer, bone metastasis, osteolytic, signal transduction

## Abstract

**Objective:**

The present study aimed to investigate the involvement of aberrant BMP8A expression in TNBC and bone metastasis.

**Methods:**

Aberrant expression of BMP8A in breast cancer was first determined by analyzing The Cancer Genome Atlas breast cancer cohort (TCGA-BRCA) and an immunohistochemical (IHC) staining of BMP8A in a breast cancer tissue microarray (TMA). Clinical relevance of deregulated BMP8A in breast cancer was assessed using Kaplan-Meier online analysis. The influence of BMP8A on cellular functions of two TNBC cell lines was assessed using *in vitro* assays. Conditional medium (CM) collected from the supernatant of hFOB cells and bone matrix extract (BME) was applied to mimic the bone micro-environment to evaluate the role played by BMP8A in bone metastasis. Correlations with both osteolytic and osteoblastic markers were evaluated in the TCGA-BRCA cohort. Expression of certain responsive genes was quantified in the BMP8A overexpression cell lines. Additionally, signal transduction through both Smad-dependent and independent pathways was evaluated using Western blot assay.

**Results:**

Compared to the adjacent normal tissues, BMP8A expression was significantly increased in primary tumors (*p* < 0.05) which was associated with shorter distant metastasis free survival (DMFS) in TNBC (*p* < 0.05). BMP8A was observed to enhance cell invasion and migration within TNBC cells. In the simulated bone milieu, both MDA-MB-231^BMP8Aexp^ and BT549^BMP8Aexp^ cells presented enhanced invasiveness. BMP8A level was strongly correlated with most osteolytic and osteoblastic markers, suggesting the potential involvement of BMP8A in bone metastasis in TNBC. Receptor activator of nuclear factor kappa-B ligand (RANKL) expression was significantly increased in BMP8A overexpressed triple-negative cell lines (MDA-MB-231 and BT549). Furthermore, enhanced phosphorylation of Smad3 and increased expression of epidermal growth factor receptor (EGFR) were observed in MDA-MB-231 cells overexpressing BMP8A.

**Conclusion:**

BMP8A was upregulated in TNBC which was associated with poorer DMFS. BMP8A overexpression enhanced the invasion and migration of TNBC cells. With a putative role in osteolytic bone metastasis in TNBC, BMP8A represents a promising candidate for further investigation into its therapeutic potential.

## 1 Introduction

TNBC is a heterogeneous group of breast cancers characterized by the absence of estrogen receptor (ER), progesterone receptor (PR), and human epidermal growth factor receptor 2 (HER2). It is more frequently seen in younger patients (less than 50 years old), accounting for 10%–20% of invasive breast cancers (Kumar and Aggarwal, 2016). Although TNBC is sensitive to chemotherapy, the prognosis of this subtype is the worst compared with other subtypes due to a lack of therapeutic targets. The follow-up study indicated that the risk of recurrence peaked 3 years post-surgery, with the incidence of metastatic relapses surpassing that of other subtypes (Kumar and Aggarwal, 2016).

Bone morphogenetic protein (BMP) signaling is actively involved in disease progression and metastasis of TNBC. For instance, BMP6 was found to promote the expression of E-cadherin in MDA-MB-231 cells, thereby facilitating cell adhesion (Yang et al., 2007). Additionally, hypermethylation of BMP6 was observed in the ER-negative breast cancer specimens, as well as in MDA-MB-231 cells (Zhang et al., 2007). BMP6 reduced the invasiveness of MDA-MB-231 cells through a downregulation of matrix metalloproteinase 1 (MMP1) at both the transcript and protein levels (Hu et al., 2016). Similarly, BMP4 has been reported to diminish the expression of MMP9 in TNBC cell lines, including MDA-MB-231 and MDA-MB-468 (Laulan and St-Pierre, 2015). In addition to regulating MMPs, BMP2 enhanced estradiol-stimulated proliferation of MDA-MB-231 cells via upregulation of ERα-36 (Wang et al., 2012).

A previous study revealed that high BMP8A expression was associated with poorer overall survival (OS) in breast cancer (Katsuta et al., 2019). To further elucidate the subtype-specific role of BMP8A and its involvement in bone metastasis in TNBC cells, which exhibit the highest incidence of bone metastasis, a lentiviral vector harboring the coding sequence of human BMP8A was also employed to overexpress BMP8A in MDA-MB-231 and BT549 cell lines. Subsequent *in vitro* cell functional analyses were conducted to investigate relevant genes associated with altered cellular functions and signaling pathways. Furthermore, the cells were also exposed to bone environment using *in vitro* models.

## 2 Materials and methods

### 2.1 Cell lines

Two TNBC cell lines, MDA-MB-231 and BT549 were used in the current study. MDA-MB-231 and BT549 cell lines were cultured in DMEM medium with 10% fetal bovine serum (FBS) and antibiotics. An osteoblast cell line, hFOB1.19 (ATCC) was grown in Ham’s F12 Medium with L-glutamine (without phenol red) containing 0.3 mg/mL G418% and 10% FBS.

### 2.2 Overexpression of BMP8A in MDA-MB-231 and BT549 cells

Lentiviral vectors encoding BMP8A (VB180207-1092dmp, Vectorbuilder, Chicago, IL, United States) and corresponding control vectors were employed to establish BMP8A overexpression and paired control cells in both MDA-MB-231 and BT549 cell lines, respectively. When the cell confluence reached 70%–80%, the cells were rinsed twice with PBS and subsequently added with fresh medium (600 µL) in each well. After adding 8 μg/mL polybrene into the wells, 400 µL of lentiviral particles were added into each well gradually, followed by a gentle mixture. The plate was incubated at 37°C with 5% CO_2_ for 24 h. Selection was conducted using 200 μg/mL hygromycin for a duration of up to 2 weeks. After a verification of the expression of BMP8A, stable cell lines were maintained in a medium containing 50 μg/mL hygromycin.

### 2.3 RNA isolation, cDNA synthesis, polymerase chain reaction (PCR), and real-time quantitative PCR (qPCR)

RNA extraction and subsequent cDNA synthesis from the cell models were undertaken strictly according to a previously described procedure (Sui et al., 2023). Following this, conventional PCR and qPCR were performed. Glyceraldehyde-3-phosphate dehydrogenase (GAPDH) was used as a housekeeping gene. Primer sequences are provided in Table 1.

**Table 1:** Primers for PCR and qPCR.

Gene	Primer name	Primer sequence (5′-3′)
GAPDH	SGF1	TGC​ACC​ACC​AAC​TGC​TTA​GC
	SGR1	GGC​ATG​GAC​TGT​GGT​CAT​GAG
	F8	GGC​TGC​TTT​TAA​CTC​TGG​TA
	R8	GAC​TGT​GGT​CAT​GAG​TCC​TT
BMP8A	SGF1	CTG​GTT​GCT​GAA​GCG​TCA​CAA​G
	SGR1	AGT​GAC​CAC​GAA​AGG​CTG​TTG​G
	F8	GCC​TCT​ATG​TGG​AGA​CTG​AG
	R8	CAC​TCC​CCC​TCA​CAG​TAA​TA
Snail	F17	CACACTGGCGAGAAGC
	ZR17	***ACT​GAA​CCT​GAC​CGT​ACA*** CTT​CTT​GAC​ATC​TGA​GTG​GG
Slug	F17	TGG​ACA​CAC​ATA​CAG​TGA​TT
	ZR17	***ACT​GAA​CCT​GAC​CGT​ACA*** GAT​CTC​TGG​TTG​TGG​TAT​GA
MMP1	SGF1	GGG​AGA​TCA​TCG​GGA​CAA​CTC
	SGR1	GGG​CCT​GGT​TGA​AAA​GCA​T
MMP2	SGF1	TGA​TCT​TGA​CCA​GAA​TAC​CAT​CG
	SGR1	GGC​TTG​CGA​GGG​AAG​AAG​TT
MMP10	SGF1	TCC​AGG​CTG​TAT​GAA​GGA​GAG​G
	SGR1	GGT​AGG​CAT​GAG​CCA​AAC​TGT​G
MMP11	SGF1	CCT​GCA​TCT​GTC​TGC​CTT​CT
	SGR1	GCTTTHHAGGATAGCAGTGC
MMP14	SGF1	CCT​TGG​ACT​GTC​AGG​AAT​GAG​G
	SGR1	TTC​TCC​GTG​TCC​ATC​CAC​TGG​T

Note: The italic sequence, ***ACTGAACCTGACCGTACA*** in the respective primers is known as the Z-sequence was used in the respective primer pairs for quantitative PCR assays.

### 2.4 Protein extraction, sodium dodecyl sulfate-polyacrylamide gel electrophoresis (SDS-PAGE), and western blot analysis

Following a protein extraction using (radio-immunoprecipitation assay) RIPA buffer, protein samples were quantified using the DC Protein Assay kit (BIO-RAD, United States). The protein samples were separated with SDS-PAGE and transferred onto polyvinylidene fluoride (PVDF) membranes using a semi-dry transferring method. The membranes were initially blocked using a solution of 10% skimmed milk powder in TBS with 0.1% Tween 20. Subsequently, they were incubated overnight with specific primary antibodies at a dilution of 1:1,000 in a solution containing 2.5% skimmed milk powder in TBS with 0.1% Tween 20 (Table 2). This was followed by incubation with the corresponding secondary antibodies at a dilution of 1:1,000 (Table 3). After washing membrane using 2.5% skimmed milk powder in TBS with 0.1% Tween 20 and 0.2% Tween 20 in TBS, protein bands were visualized using a chemiluminescence detection kit (Luminate Forta Western HRP substrate, Cat. No. WBLUF0500, Merck-Millipore, Hertfordshire, United Kingdom) and a UVITech Imager (UVITech Inc., Cambridge, United Kingdom) (Zeng et al., 2022).

**Table 2:** Primary antibodies applied in the present study.

Name	Species	kDa	Supplier	Product code	Dilutions
Anti-BMP8A	Rabbit	43	Abcam	ab60290	1:1,000
Anti-GAPDH	Mouse	37	Santa Cruz	sc-32233	1:5,000
Anti-Snail1	Mouse	29	Santa Cruz	sc271977	1:1,000
Anti-Slug	Mouse	29.9	Santa Cruz	sc166476	1:1,000
Anti-AKT1	Mouse	55.7	Santa Cruz	sc5298	1:1,000
Anti-P-AKT-1	Mouse	55.7	Santa Cruz	sc81433	1:1,000
Anti-JNK	Rabbit	46	Santa Cruz	sc-571	1:1,000
Anti-P-JNK	Mouse	46	Santa Cruz	sc6254	1:1,000
Anti-P38	Mouse	38	Santa Cruz	sc7972	1:1,000
Anti-P-ERK	Mouse	44	Santa Cruz	sc7383	1:1,000
Anti-ERK	Mouse	44	Santa Cruz	sc514302	1:1,000
Anti-Vimentin	Mouse	53.7	Santa Cruz	sc66002	1:1,000
Anti-P-Smad1/5/8	Rabbit	53	Sigma	ab3848-1	1:1,000
Anti-RANKL	Rabbit	35.5, 27.7, 30.5	Santa Cruz	sc9073	1:1,000
Anti-EGFR	Mouse	134.3	Santa Cruz	sc71034	1:1,000
Anti-CTSK	Mouse	37	Santa Cruz	sc48353	1:1,000
Anti-PTHrP	Rabbit	20.2	Santa Cruz	sc20728	1:1,000
Anti-VEGF	Goat	27, 25, 24	Sigma	V6627	1:1,000
Anti-RANK	Rabbit	66, 56.4, 36.3	Santa Cruz	sc9072	1:1,000
Anti-P-Smad3	Rabbit	48.1	Santa Cruz	sc130218	1:1,000

**Table 3:** Secondary antibodies.

Name	Species	Supplier	Product code
Anti-mouse IgG	Rabbit	Sigma	A-9044
Anti-rabbit IgG	Goat	Sigma	A-9169
Anti-goat IgG	Rabbit	Sigma	A-5420

### 2.5 *In vitro* cell proliferation assay

Three thousand MDA-MB-231 and BT549 cells in 200 μL medium were seeded in three 96-well plates and incubated at 37°C with 5% CO_2_ for 1, 3, 5 days respectively. Each cell line had six replicates on each plate. The cells were fixed using 4% formalin and stained with crystal violet. After dissolving the crystal violet stain using 10% acetic acid, absorbance was determined using a spectrophotometer (BIO-TEK, Elx800, United Kingdom) at the wavelength of 540 nm.

### 2.6 *In vitro* cell adhesion assay

Before conducting the cell adhesion assay, 96-well plates were pre-coated with 5 µg Matrigel at a concentration of 50 µg/mL. Subsequently, 30,000 cells were seeded onto the bottom of each well. The cells were incubated for 40 min at 37°C in a 5% CO_2_ atmosphere to adhere to the matrix protein coating the bottom of the plate. Following incubation, they were fixed in formalin and subsequently stained with crystal violet. After adding 100 µL of 10% acetic acid into the wells, the absorbance was measured at the wavelength of 540 nm. Each cell line was represented in six replicates per experiment.

### 2.7 Cell invasion assay

Thirty thousand cells (MDA-MB-231 and BT549) were seeded per transwell insert which were precoated with 50 μg Matrigel. After 3 days’ incubation at 37°C with 5% CO_2_, the insert and control well were fixed and further stained with crystal violet. After adding 100 µL of 10% acetic acid into the wells, the absorbance was read to determine the levels of cell invasion within control and BMP8A overexpression cells.

### 2.8 Cell migration assay

Cells (MDA-MB-231 and BT549) were seeded on a 24-well-plate followed by an overnight culture to form a monolayer. Wounds were made using a fine pipette tip. Migration of cells was monitored using the EVOS (auto imaging system, Thermo Fisher Scientific, Waltham, MA United States) over a course of up to 6 h. ImageJ software was applied to determine the migrated distance.

### 2.9 Zymography

Following quantification of cell numbers, the same amount of control and BMP8A overexpression MDA-MB-231 cells were seeded onto a 6-well-plate to achieve 90% confluence overnight. Fresh medium (400 µL) was added into the well and collected after 8 h incubation. Non-reducing sample buffer containing 4% SDS, 20% glycerol, 0.01% bromophenol blue, and 125 mM Tris-HCl (Tris hydroxymethyl aminomethane hydrochloride) with pH 6.8 was used to prepare the protein samples for subsequent zymography analyses. After an electrophoresis in the SDS-PAGE with gelatin. The gel was then washed with a washing buffer (containing 2.5% Triton X-100 + 0.02% NaN3) and incubated with an incubation buffer (containing 50 mM Tris-HCl, 5 mM CaCl2, 0.02% NaN3, pH 8.0) at 37°C overnight. The gel was then stained with Coomassie brilliant blue and washed with a destaining buffer (10% acetic acid and 25% ethanol) before capturing images of the developed bands.

### 2.10 Preparation of BME

BME was previously prepared at the host laboratory (Davies, 2008). In brief, femur bone tissues were collected from patients undergoing total hip replacements at the Trauma and Orthopaedic Department of University Hospital of Wales and Llanddough Hospital. The collection was conducted subsequent to obtaining written informed consent from the donors and was carried out in strict compliance with a protocol that had received ethical approval from the Bro Taf Research Ethics Committee. The fragments were further processed using a Bioruptor (Diagenode, Seraing, Belgium) before adding PBS buffer. After a centrifugation at 1600 rpm for 10 min, supernatant was collected. Total protein content of the BME was then quantified using a Bio-Rd DC protein assay kit (Bio-Rad Laboratories, Hemel Hempstead, United Kingdom) before being standardized to 2 mg/mL. The BME was stored at −80°C for further use.

### 2.11 Collection of the CM from hFOB cells

Once cell confluence of hFOB cells reached 80% in a T25 flask, the culture medium was replaced after rinsing twice with PBS. Following an incubation for 6 h, the culture medium was collected, filtered to remove the debris and stored at −80°C for further use.

### 2.12 IHC of BMP8A in breast cancer TMA

The IHC staining of BMP8A was performed on a TMA (BC 081120f, Biomax, United States) using anti-BMP8A/OP-2 antibody (ab60290, Abcam) following a previously reported procedure (Ye et al., 2010). An assessment of the IHC staining was conducted by a pathologist (TW) according to staining intensity and percentage of positive cells. The staining intensity was scored as: 1- weak; 2-medium; 3-strong, whereas the percentage was assessed as follows: 0- less than 1% of the cancer cells being positive, 1- 1%–40% cells being positive; 2- 40%–70% were positive; 3- 70%–100% being positive.

### 2.13 Statistical analysis

One-way ANOVA test was employed for statistical analysis of data comprising multiple groups, whilst non-normally distributed data were assessed using Mann-Whitney tests and Kruskall-Wallis Test. Normally distributed experimental data were analyzed using t-tests. Kaplan-Meier survival analysis was performed for BMP8A in breast cancer using an online platform (www.kmplot.com/) (Gyorffy, 2021). Correlation coefficients between different genes were evaluated using Spearman test. *P*< 0.05 was regarded as statistically significant. One-way ANOVA, Mann-Whitney test, t-test and Spearman correlation test were performed using SPSS (version27, SPSS Inc., Chicago, IL, United States).

## 3 Results

### 3.1 Aberrant expression of BMP8A in TNBC and the clinical implication

**FIGURE 1:**
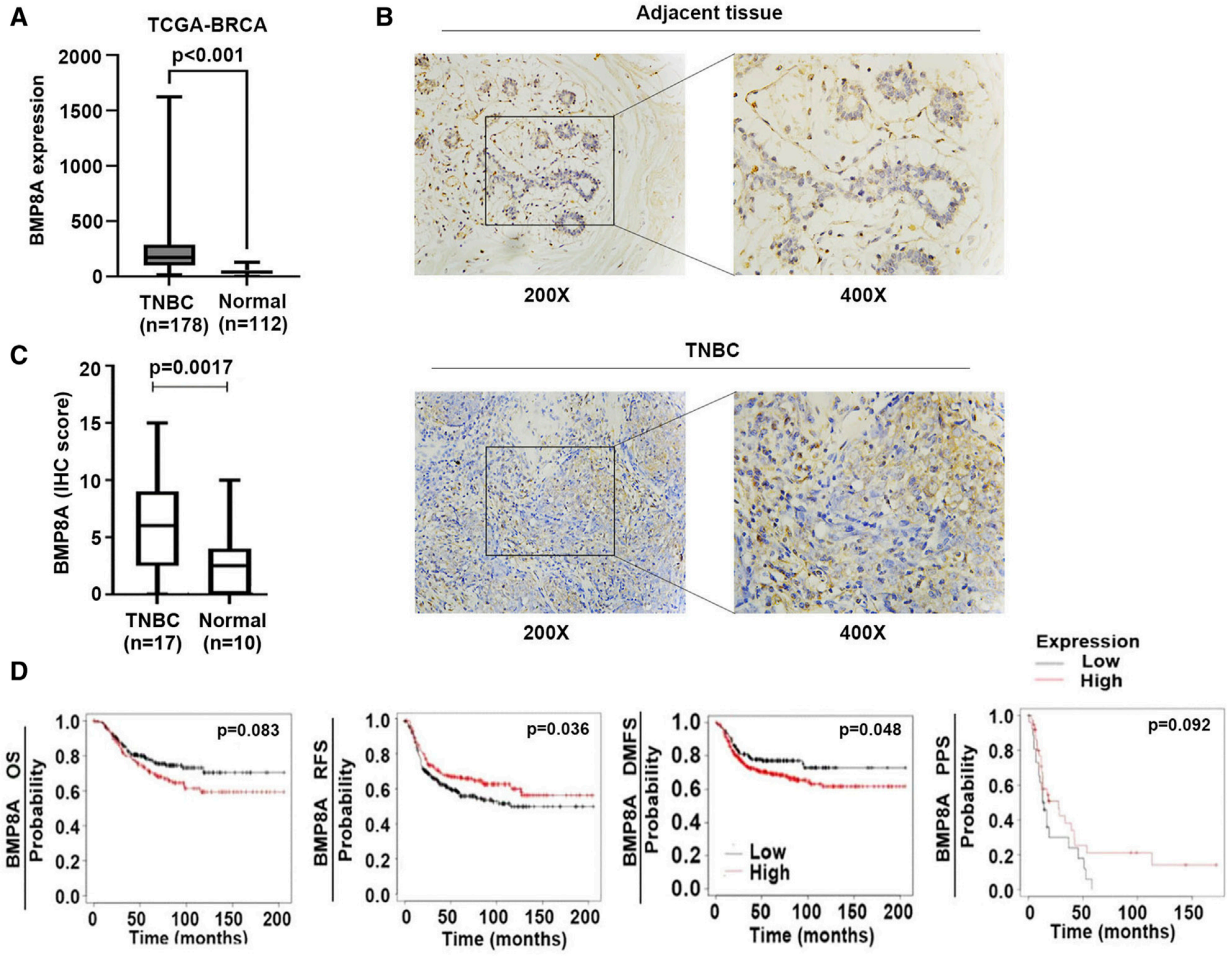
Aberrant BMP8A expression in TNBC and the clinical implication. **(A)** Expression of BMP8A in the TNBC tumors was compared with normal breast tissues in the TCGA-BRCA cohort using Mann-Whitney test. Shown are normalized RSEM (RNA-Seq by Expectation-Maximization) as transcripts per million (TPM). **(B)** IHC staining showed the differential expression and distribution of BMP8A in TNBC. **(C)** Shown are IHC scores of BMP8A in TNBC tumors in comparison with normal controls which was analyzed using the Mann-Whitney test. **(D)** Clinical implication of BMP8A in TNBC regarding patients’ survival was analyzed using the Kaplan-Meier Plotter (www.KMPlot.com). Shown are association with OS, RFS, DMFS and PPS.

A significantly elevated BMP8A expression was presented in the TNBC tumors in comparison with the normal tissues according to an analysis of the TCGA-BRCA (Figure 1A). The IHC staining also showed an increased staining of BMP8A in TNBC samples compared with adjacent normal tissues (Figure 1B, C). BMP8A staining was mainly distributed in the cytoplasm, some staining was seen in the cell nuclei, and some staining was also observed in carcinoma stroma such as fibrocytes and immune cells (Figure 1B).

The increased expression of BMP8A was associated with poorer DMFS in patients with TNBC (*p* = 0.048), and shorter OS but the latter did not reach a statistically significant level (*p* = 0.083) (Figure 1D). However, low expression of BMP8A was found to be associated with poor relapse free survival (RFS) in those patients with TNBC (*p* = 0.036) which was also likely to be associated with poor post-progression survival (PPS) (*p* = 0.092) (Figure 1D).

### 3.2 Role of BMP8A in TNBC cells *in vitro*

**FIGURE 2:**
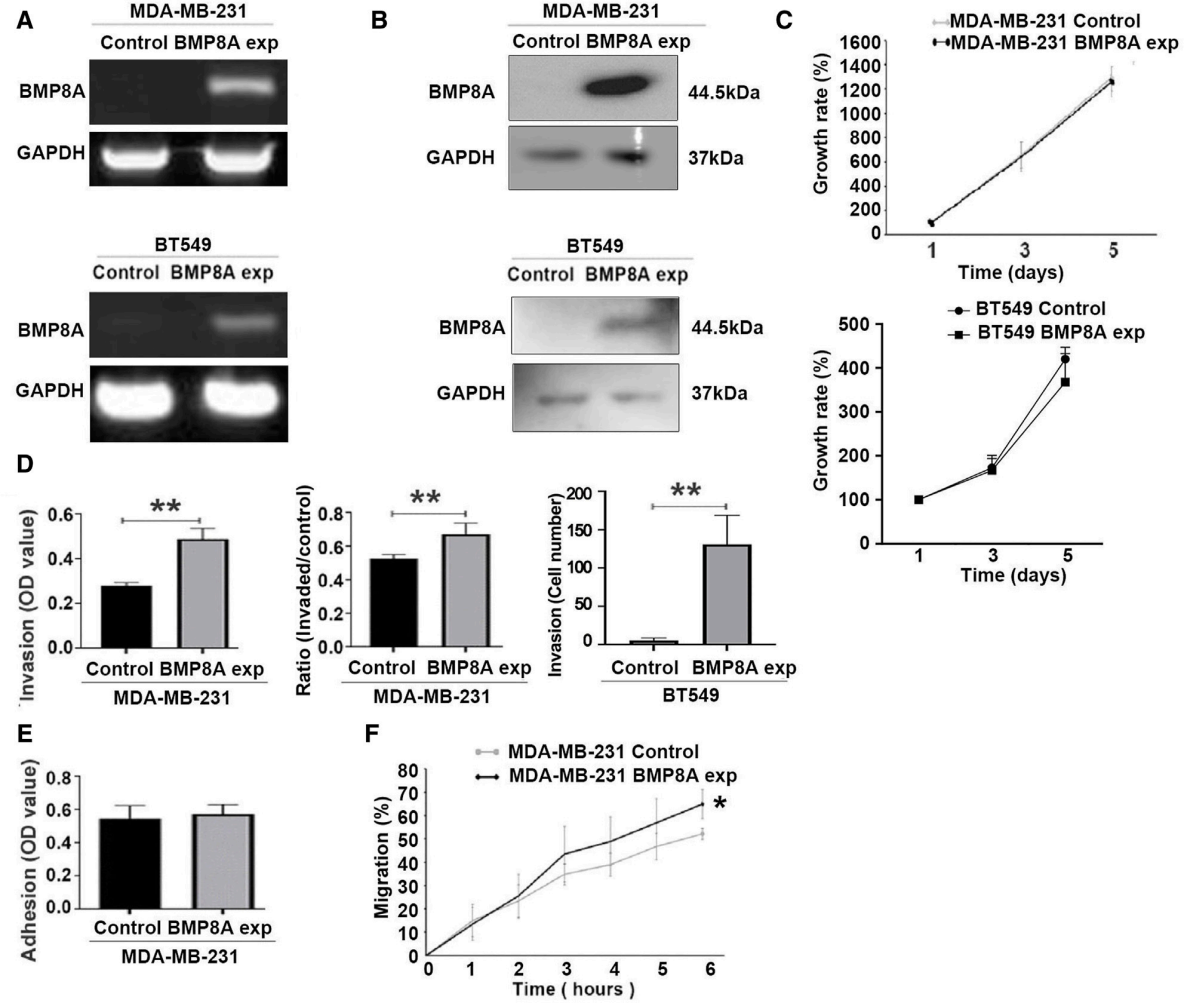
Influence of BMP8A overexpression on *in vitro* cellular functions of MDA-MB-231 and BT549 cells. BMP8A overexpression in MDA-MB-231 and BT549 cell lines were verified using conventional PCR **(A)**, and Western blot **(B)**. **(C)** A colorimetric proliferation assay using crystal violet was employed for the evaluation of proliferation affected by BMP8A overexpression. **(D)** Effect on invasiveness of MDA-MB-231 and BT549 cells was determined using the transwell invasion assay. **(E)** Adhesion assays were conducted to assess adhesion of MDA-MB-231 cells to Matrigel. **(F)** Influence of BMP8A on migration of MDA-MB-231 cells was determined using the migration assay (wound assay). Closure of wounds as percentages (%) was determined over a time course up to 6 h. Shown in the bar graphs are mean and standard deviation (SD). **p < 0.01, *p < 0.05.

Increased expression of BMP8A in the transduced MDA-MB-231 and BT549 cells was confirmed with conventional PCR (Figure 2A) and Western blots (Figure 2B).

Influence of BMP8A overexpression on cellular functions was assessed using *in vitro* cellular function assays including proliferation, adhesion, invasion, and migration. After incubation up to 5 days, no significant change of cell proliferation was observed in either MDA-MB-231 and BT549 cells following the overexpression of BMP8A (Figure 2C). However, BMP8A overexpression resulted in a significant increase of invasiveness for both cell lines (Figure 2D). No significant change was observed in the adhesion of MDA-MB-231^BMP8Aexp^ cells compared to the control cells (Figure 2E). In addition to the enhanced invasion, cell migration was also increased significantly in MDA-MB-231 cells with BMP8A overexpression (Figure 2F).

### 3.3 Molecular mechanism of enhanced cell invasion in BMP8A upregulated MDA-MB-231 and BT549 cells

**FIGURE 3:**
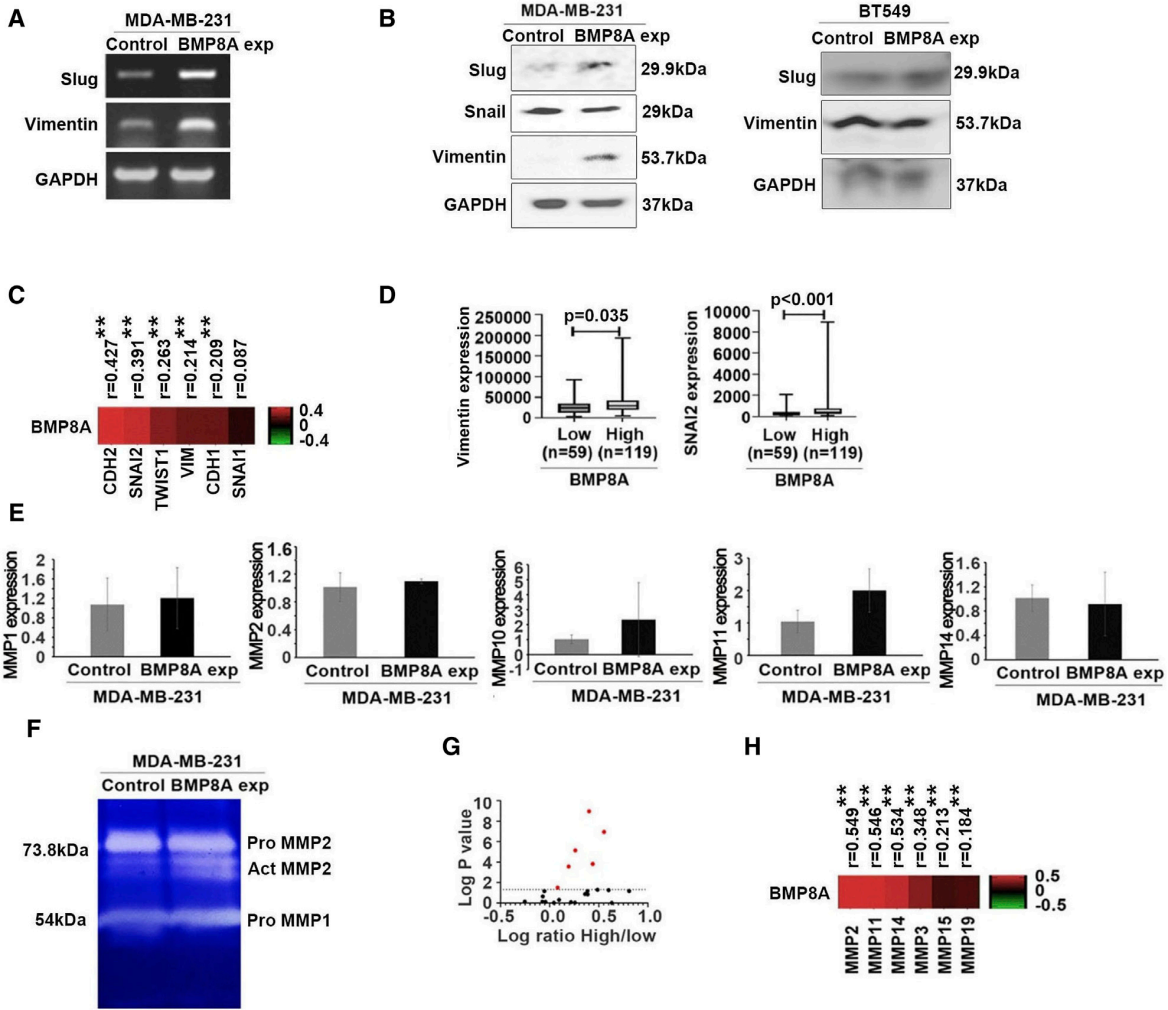
Impact of BMP8A on EMT and BMP8A induced differential MMPs in MDA-MB-231 and BT549 cell lines. **(A)** Differently expressed Slug and vimentin were detected using conventional PCR **(A)** and Western blot **(B)**. **(C)** Heatmap shows the correlation efficiency between BMP8A and EMT markers in the TCGA-BRCA cohort. Spearman test was employed for the correlation analysis. **(D)** Expression of VIM and SNAI2 in the TCGA-BRCA cohort TNBC tumors was analyzed according to their expression of BMP8A (cut-off value = 128.1) using Mann-Whitney test. Shown are TPM of normalized RSEM. **(E)** Expressions of MMPs in MDA-MB-231 cells were determined using qPCR. Shown are changes in folds using the ∆∆CT method. **(F)** Expression and activity of MMPs were determined using zymography. **(G)** Differential expressions of MMPs in the TNBC tumors of the TCGA-BRCA cohort were analyzed using Mann-Whitney test by a separation of tumors regarding their expression of BMP8A with the cut-off value of 128.1. Shown in volcano plot is log value of change in folds for the TNBC tumors with high BMP8A against those tumors with low expression of BMP8A. Red dots are those MMPs with statically significant changes (*p* < 0.05). **(H)** Heatmap shows correlations between BMP8A and those differentially expressed MMPs (the red points in panel G) that were analyzed using Spearman test. Shown in the bar graphs are mean and SD. *p < 0.05 and **p < 0.01.

To explore the influence of BMP8A on epithelial-mesenchymal transition (EMT), one of the pivotal processes affecting cell motility, expression of the EMT markers including Snail, Slug, vimentin and cadherin 2 (CDH2, also known as N-cadherin) were determined for their expression at both mRNA and protein levels. Increased expression of Slug was noted in MDA-MB-231^BMP8Aexp^ cells (Figure 3A, B) and BT549^BMP8Aexp^ cells (Figure 3B) at mRNA and protein levels, respectively. Vimentin was upregulated by BMP8A in MDA-MB-231 in which vimentin was barely detectable, while BMP8A had little impact on the expression of vimentin in BT549 cells which had a high expression of this protein. A correlation between BMP8A and the EMT markers in the TNBC tumors was then analyzed using RNA sequencing data from the TCGA-BRCA cohort. BMP8A transcript levels were positively correlated with most EMT markers including Snai2 (Slug), Twist, vimentin and CDH2 (N-cadherin), but not Snail (Figure 3C). The TCGA-BRCA TNBC tumors were then separated into two groups according to their expression of BMP8A with a cut-off value of 128.1. Expression of Snai2 (Slug) and vimentin was also significantly higher in the TNBC tumors with high expression of BMP8A (Figure 3D).

The influence of BMP8A on the enhanced invasiveness in MDA-MB-231 cells was also investigated by determining the expression of several matrix metalloproteases (MMPs). Although no significant change was seen for MMP2 at transcript levels (Figure 3E), enhanced MMP2 activity was observed in MDA-MB-231^BMP8Aexp^ cells compared to the control cells according to a gelatin zymography analysis (Figure 3F). No significant alterations were observed in the expression levels of other MMPs in the MDA-MB-231^BMP8Aexp^ cells compared to the control cells.

Expression analysis of MMPs in TNBC tumors with elevated BMP8A levels was conducted and compared to TNBC tumors with lower BMP8A expression, paralleling previous analyses of Snai2 and vimentin in TCGA TNBC tumors. A number of MMPs exhibited higher expressions in the TNBC tumors with more abundant expression of BMP8A, including MMP2, MMP3, MMP11, MMP14, MMP15 and MMP19. Those MMPs also have a significantly positive correlation with BMP8A in the TNBC tumors of the TCGA cohort (Figure 3G, H). MMP2 was identified as the principal molecule associated with BMP8A, exhibiting a correlation coefficient of 0.549 (*p* < 0.01).

### 3.4 Involvement of BMP8A in bone metastasis of TNBC

**FIGURE 4:**
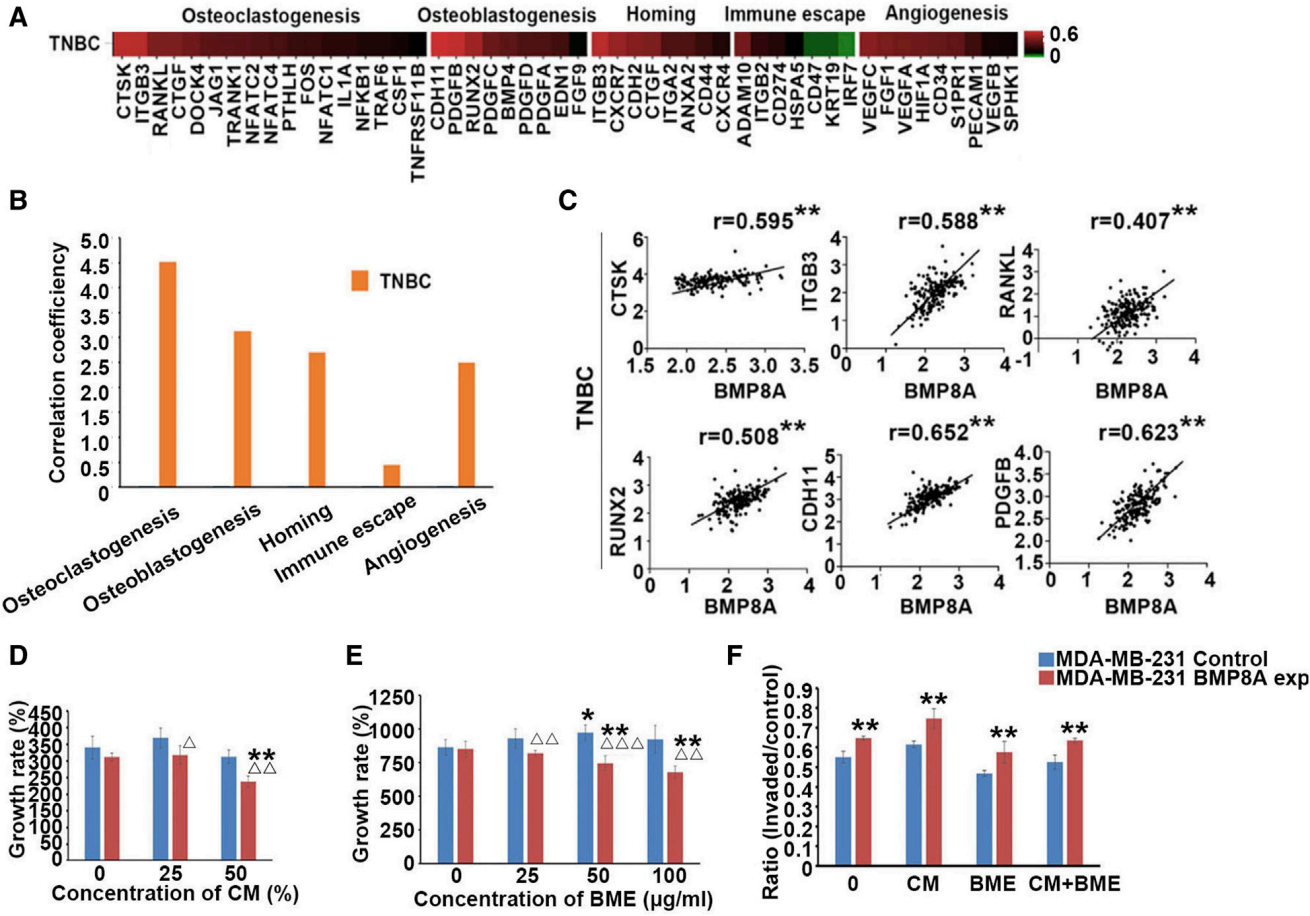
Involvement of BMP8A in bone metastasis of TNBC. **(A)** Heatmap shows the correlation between BMP8A and bone metastasis related markers in the TNBC tumors of the TCGA-BRCA cohort according to an analysis of their transcripts levels using Spearman test. **(B)** Correlation coefficients of BMP8A with those markers were summed and presented as bar graph. **(C)** Scatter plots show the correlations between BMP8A and the most correlated markers. **present *p* < 0.01. **(D)** Impact of BMP8A on cell growth in exposures to different concentrations of CM collected from hFOB cells was determined after a 3-day incubation. **(E)** Influence of BME on the cell proliferation was also determined after an incubation of 5 days. **(F)** Altered cell invasion following exposures to different *in vitro* bone environments was also determined as shown in ratios of invaded cells. Shown in the bar graphs are mean and SD. Δ *p* < 0.05, ΔΔ *p* < 0.01 and ΔΔΔ *p* < 0.001, VS. MDA-MB-231^control^; *p < 0.05 and **p < 0.01 VS. corresponding untreated MDA-MB-231^control^ and MDA-MB-231^BMP8Aexp^.

To further investigate the role of BMP8A in bone metastasis, a common complication of BC, particularly within TNBC subtype, this study examined the correlations between BMP8A and key biomarkers associated with bone metastases, including osteoblastogenesis, osteoclastogenesis, homing, immuno-escape, and angiogenesis in TCGA-BRCA cohort. BMP8A was highly correlated with most osteolytic and osteoblastic markers in TNBC subtype (Figure 4A). Additionally, significant correlation coefficients were observed with markers related to homing and angiogenesis in TNBC. To provide a comprehensive overview, the correlation coefficients were summed up to assess an overall relationship between BMP8A and these markers of bone metastasis (Figure 4B), revealing high correlations with osteoclastic and osteoblastic markers in TNBC. Correlations of the top three osteoclastic Cathepsin K (CTSK), integrin β3 (ITGB3), and RANKL and osteoblastic markers Runt-Related Transcription Factor 2 (RUNX2), cadherin 11 (CDH11) and platelet derived growth factor B (PDGFB) with BMP8A was presented in a scatter plot format. As demonstrated, correlation scores were markedly high in TNBC (Figure 4C), which indicated the potential involvement of BMP8A in promoting bone metastasis in TNBC.

To investigate the involvement of BMP8A in bone metastasis, BME and CM collected from the hFOB cells were utilized to mimic the bone environment. Six thousand MDA-MB-231 cells were seeded in the 96-well plate and treated with CM at different concentrations (25% and 50%). After a 3-day incubation, proliferation in MDA-MB-231^BMP8Aexp^ cells was significantly reduced compared with the control cells under a culture with 25% of the CM (*p* < 0.05). With 50% CM, decreased cell growth was even more obvious when compared with both control cells and MDA-MB-231^BMP8Aexp^ cells cultured in normal medium (*p* < 0.01) (Figure 4D). hFOB CM exhibited an inhibitory effect on cell proliferation of MDA-MB-231 cells when BMP8A was overexpressed, which was not observed in control cells.

In addition to the tests using the hFOB CM, proliferation assays were also conducted by exposing the MDA-MB-231 cells to BME. After 5 days of culture, the proliferation of MDA-MB-231^BMP8Aexp^ cells was reduced significantly when exposed to BME (*p* < 0.01), whereas no reduction was seen in the control cells treated with the BME. Instead, an increase in proliferation was noted in the MDA-MB-231^control^ cells when they were cultured in a medium containing 50 μg/mL of BME (Figure 4E).

Invasion of the MDA-MB-231 cells was assessed *in vitro* after exposure to the BME. Thirty thousand cells were seeded into the plate with CM (50%) and BME (50 μg/mL). After 3 days, significantly enhanced cell invasion was found in MDA-MB-231^BMP8Aexp^ cells compared to the corresponding control group (Figure 4F).

### 3.5 Expression of osteolytic/osteoblastic markers in MDA-MB-231 and BT549 cells with BMP8A overexpression

**FIGURE 5:**
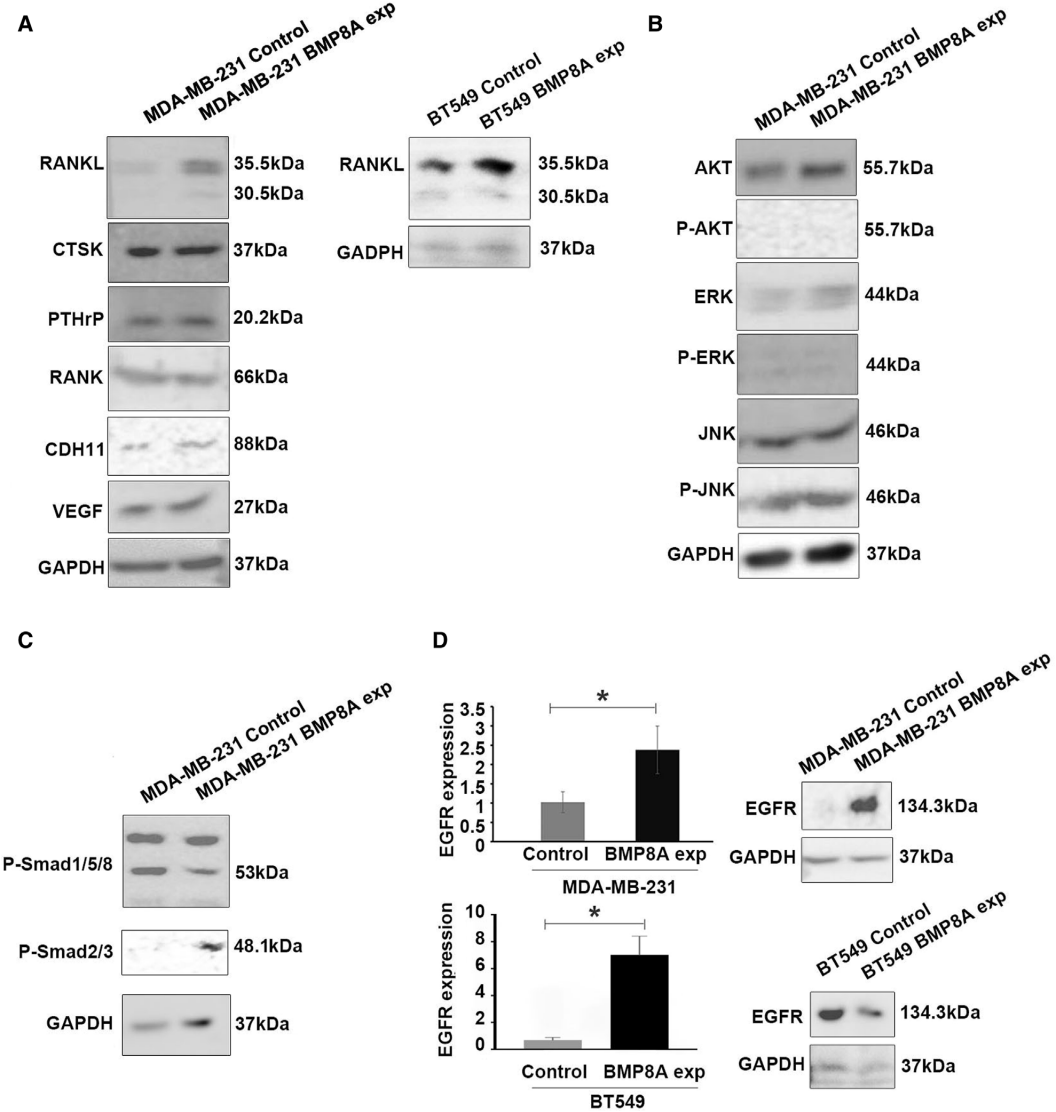
The molecular mechanism of BMP8A induced bone metastasis and BMP8A signaling in the TNBC cell lines. **(A)** Some osteolytic and osteoblastic markers were examined in the TNBC cell line models with BMP8A overexpression using Western blots. Involvement of Smad independent **(B)** and Smad dependent **(C)** signaling in MDA-MB-231^BMP8Aexp^ cells was assessed using Western blot. **(D)** EGFR expression was determined using both qPCR and Western blot. Shown in the bar graphs are mean and SD for changes in folds that were calculated using the ΔΔCT method. *represents *p* < 0.05.

To assess the possible role of BMP8A in bone metastasis, the expression of promising biomarkers including osteolytic, osteoblastic, and angiogenesis were determined in MDA-MB-231 cells. From the present research, increased RANKL expression was found in MDA-MB-231^BMP8Aexp^ cells, which strongly indicated that BMP8A may take part in the bone metastasis via RANKL - Osteoprotegerin (OPG) signaling (Figure 5A). No differential expression of CTSK, parathyroid hormone-related protein (PTHrP), receptor activator of nuclear factor kappa B (RANK), and vascular endothelial growth factor (VEGF) were detected (Figure 5A). CDH11, a pivotal mediator of osteoblastogenesis, was increased in the cells with BMP8A overexpression (Figure 5A). To further determine the possible role of BMP8A in bone metastasis in TNBC, expression of RANKL was also investigated in BT549 cells with upregulated BMP8A expression, where elevated RANKL was observed (Figure 5A).

### 3.6 Influence of BMP8A on smad independent and smad dependent signaling

The influence of BMP8A on Smad independent signaling, including protein kinase B (AKT) and mitogen-activated protein kinase (MAPK) signaling including extracellular signal-regulated kinase (ERK), Jun N-terminal kinase (JNK) was also determined. Although upregulated AKT and ERK expression were observed in MDA-MB-231 cells with BMP8A overexpression, phosphorylated AKT appeared to be undetectable, and no differences were found in phosphorylated ERK. Notably, subtly enhanced JNK phosphorylation was seen in MDA-MB-231^BMP8Aexp^ cells (Figure 5B). Differing from classic Smad1/5/8 activation in BMP signaling, phosphorylation of Smad3 was observed in MDA-MB-231^BMP8Aexp^ cells in which a reduced Smad1/5/8 phosphorylation was also detected (Figure 5C).

### 3.7 Differential expression of EGFR in the BMP8A overexpression cell line models

Differential expressions of EGFR were investigated in BMP8A-modified cell models in the present study (Figure 5D) using qPCR and Western blot analysis. Increased EGFR expression was observed in MDA-MB-231^BMP8Aexp^ cells at both mRNA and protein levels. However, a similar change of EGFR was not seen in BT549^BMP8Aexp^ cells at both mRNA and protein levels although EGFR transcripts appeared to be increased in the BT549^BMP8Aexp^ cells.

## 4 Discussion

BMP signaling has been evident in the regulation of both proliferation and invasion in TNBC cells. BMP2 exerts an inhibitory effect on cell growth and induces cellular apoptosis of MDA-MB-231 cells (Chen et al., 2012). By upregulating the expression of microRNA-192, BMP6 was found to induce cell cycle arrest in MDA-MB-231 cells (Hu et al., 2013). Cell growth in BMP9 overexpressed MDA-MB-231 cells was significantly inhibited compared to the control cells due to a downregulation of PI3K(Phosphatidylinositol-3-kinase)/AKT signaling pathway (Li et al., 2018). Tumor invasion and migration of TNBC cells were suppressed by interfering BMP signaling (Di et al., 2019). Besides the inhibition on the cell growth, BMP6 was also found to suppress the metastasis of MDA-MB-231 cells by downregulating the expression of MMP1 (Hu et al., 2016) and upregulating E-cadherin (Yang et al., 2007). Cell growth, invasion, and motility in MDA-MB-231 BMP10 overexpression cells were significantly suppressed compared with the control cells (Ye et al., 2010). However, the impact of BMP8A in TNBC subtype remains largely unknown.

The present research was the first study to examine the influence of BMP8A on cellular functions of TNBC cells. *In vitro* cellular function tests showed that BMP8A promoted invasion and migration of both MDA-MB-231 and BT549 cells. This is in line with the finding by bioinformatic analysis that higher expression of BMP8A in TNBC was associated with poorer DMFS, as both invasion and migration are essential for the spread of the disease. Along with this finding, further research on BMP8A mediated cell invasion was performed. As no significant difference was seen in the cell adhesion, the present study focused on the impact of BMP8A on MMPs and EMT transcript factors. BMP8A overexpression resulted in increased expression of specific MMPs including MMP2 and transcription factor Slug, a critical factor in EMT factor. These findings suggest that BMP8A-mediated regulation of MMPs and EMT could contribute to the promoted invasion in TNBC.

In addition to their direct regulation of proliferation, migration, and invasion of cancer cells, BMP signaling also exerts fundamental effects during the osteogenic differentiation and homeostasis by regulating the balance of osteoblasts and osteoclasts. BMPs, such as BMP-2, BMP-4 and BMP-7 have been proven to promote the differentiation of the mesenchymal stem cells (MSCs) into osteoblasts (Alarmo and Kallioniemi, 2010; Carreira et al., 2014). BMPs can impact the maturation and the activation of osteoclasts through RANKL - OPG pathway (Yahiro et al., 2020). In osteolysis caused by osteoclasts, BMPs in the bone matrix are released, which could regulate the expression of CX43/GJA1 through the BMP signaling pathway to interact with osteoblast to adjust the mineralization (Shi et al., 2016).

Since BMP8A played a pivotal role in the progression of breast cancer, and breast cancer is well known as a disease prone to bone metastasis, the role of BMP8A in the bone metastasis of BC was explored in the current study. To assess the potential role BMP8A involved in bone metastasis in BC, bioinformatical analysis in the TCGA-BRCA cohort was performed. From the analysis, BMP8A was highly correlated with the biomarkers being profound in the process of bone metastasis, especially osteoblastic factors (RUNX2, CDH11 and PDGFB); and osteolytic factors (CTSK, ITGB3 and RANKL), which strongly indicated the promising role of BMP8A in the bone metastasis of BC in TNBC. The influence of BMP8A on cellular functions in the bone environment including cell proliferation and invasion was further evaluated. Following exposure to *in vitro* bone environment, including the CM from hFOB cells and BME, the proliferation of BMP8A overexpressing cells was significantly reduced compared with control cells. The mechanism for the suppressed cell proliferation of MDA-MB-231^BMP8Aexp^ is yet to be elucidated. As in the present study, enhanced cell invasiveness was found in MDA-MB-231 and BT549 with BMP8A overexpression and was even more obvious when MDA-MB-231 cells were exposed to the *in vitro* bone environment. Preliminary investigations aiming to identify possible responsive genes for TNBC bone metastasis were undertaken and the expression profiles of a panel of osteolytic, osteoblastic and angiogenesis markers were determined. Increased RANKL and CDH11 expressions were seen in the MDA-MB-231^BMP8Aexp^ cells and increased RANKL was found in BT549^BMP8Aexp^ cells. RANKL-OPG signaling is renowned for its importance in osteoclastogenesis, whilst CDH11 is an important molecule for the activation of the osteoblast, being critical for cancer cells to colonize the bone.

Apart from estrogen receptors and HER2, EGFR also plays an important role in breast cancer. A study in the *drosophila* embryo showed that BMP signal response was inversely modulated by EGFR signaling (Deignan et al., 2016). In breast cancer, high EGFR expression is found in TNBC subtype, however, analysis of the TCGA-BRCA cohort reveals that BMP8A expression is lower in TNBC compared to the other three subtypes. Recent reviews have highlighted evident crosstalk between EGFR and BMP signaling pathways. (Sun et al., 2020). Enhanced EGFR activity has been shown to modulate the activity of BMP downstream signaling molecules such as MAPK and PI3K/Akt to facilitate tumor invasion and motility (Barr et al., 2008). BMP may modulate EGFR signaling via a regulation of PTEN (phosphatase and tensin homolog deleted on chromosome ten) and PI3K/Akt (Zhang et al., 2006). In the present study, significantly increased EGFR expression was seen in MDA-MB-231^BMP8Aexp^ cells and elevated EGFR transcripts were found in BT549^BMP8Aexp^ cells which indicated an enhanced EGFR signaling may also occur in TNBC cells as a result of BMP8A overexpression. Activation of EGFR can promote cell migration and invasion in ameloblastoma cells by enhancing MMP2 and MMP9 activity (da Rosa et al., 2014). In our study, BMP8A promoted invasion and upregulated MMP2 in MDA-MB-231 cells. It suggests that BMP8A may promote the invasiveness of TNBC cells through dual mechanisms including both BMP signaling and the regulation of EGFR which are yet to be fully evaluated.

Enhanced cell invasion and migration were seen in MDA-MB-231^BMP8Aexp^ cells together with increased expression of Slug and MMP2. Promoted cell invasion was also revealed in BT549^BMP8Aexp^ cells with upregulated Slug expression. This suggests that BMP8A promotes invasiveness and migration of TNBC cells through a regulation of both EMT and MMPs. EGFR, being frequently upregulated in TNBC tumors, can promote both invasion and EMT through both MAPK and Akt pathways (Hardy et al., 2010). Additionally, EGFR signaling is closely related to tumor progression and survival (Masuda et al., 2012). In the present study, significantly elevated EGFR expression and enhanced Smad3 activation was also seen in MDA-MB-231^BMP8Aexp^ cells. It appears that upregulated BMP8A in MDA-MB-231 cells can promote the expression of MMPs and EMT, in which the exact role EGFR signaling and Smad2/3 signaling play is yet to be determined by blocking either EGFR/HER2 or BMP receptors with specific inhibitors.

Preliminary investigation of BMP8A in the bone metastasis of BC was performed in the current study. Following exposure to a mimicked bone environment, significantly reduced cell growth and increased invasiveness were observed in MDA-MB-231^BMP8Aexp^ cells. Additional research revealed upregulated RANKL expression in MDA-MB-231^BMP8Aexp^ and BT549^BMP8Aexp^cells. BMP signaling was found to downregulate ERK signaling to induce tumor cell quiescence in the bone environment (Sosa et al., 2011), which is in line with the current findings. The invasiveness of MDA-MB-231^BMP8Aexp^ cells was further enhanced while they were exposed to the *in vitro* bone environment. Increased RANKL expressions was also found in MDA-MB-231^BMP8Aexp^ and BT549^BMP8Aexp^ cells. RANKL-OPG signaling is renowned for osteoclastogenesis, activated RANKL/ERK signaling pathway is associated with activation of osteoclasts and subsequent bone loss (Zhang et al., 2022), whilst CDH11 is a key molecule for the activation of osteoblasts, inducing the premetastatic niche for bone colonization of breast cancer (Li et al., 2022). This indicates that BMP8A may act as an important molecule to mediate the osteolytic bone metastasis and the initiation of the osteoblastogenesis in TNBC through the RANKL signaling pathway. Therefore, BMP8A demonstrates significant prognostic and therapeutic potential for managing bone metastasis in TNBC. However, this still needs to be fully investigated to elucidate the exact role of BMP8A in the spread of breast cancer cells to the bone and their subsequent colonization.

BMP8A presents contrasting roles for bone metastasis in MDA-MB-231 cells. Cell growth was significantly suppressed whereas cell invasion was promoted while exposed to the bone environment *in vitro* for MDA-MB-231^BMP8Aexp^. On the other hand, RANKL (osteolytic molecule) and CDH11 (osteoblastic molecule) expression were significantly increased in MDA-MB-231^BMP8Aexp^. Further experimentation, encompassing both *in vitro* and *in vivo* metastatic models, is imperative to elucidate the role of BMP8A in bone metastasis. However, there are still limitations using the overexpression cell models in the present study, which may not fully mimic natural expression levels of BMP8A in TNBC. Signal transductions are yet to be fully investigated to shed light on BMP8A-promoted invasion by assessing receptors involved.

Taken together, upregulated BMP8A expression is correlated with high incidence of distant metastases in TNBC subtype. BMP8A can promote invasiveness of TNBC cells in which MMP2 and EMT are involved. In TNBC, BMP8A acts as an important molecule to induce osteolytic bone metastasis through EGFR/RANKL signaling pathway, highlighting its potential as a therapeutic target for bone metastases originating from TNBC tumors. Anti-BMP8A neutralizing antibodies, small inhibitors targeting BMP8A or BMP receptors, and BMP antagonists warrant further investigation.

## Figures and Tables

**Table:** 

**AKT**	Protein kinase B
**BME**	Bone matrix extract
**BMP8A**	Bone morphogenetic protein 8A
**CDH11**	Cadherin 11
**CDH2**	Cadherin 2
**CM**	Conditional medium
**CTSK**	Cathepsin K
**DMFS**	Distant metastasis free survival
**EGFR**	Epidermal growth factor receptor
**EMT**	Epithelial-mesenchymal transition
**ER**	Estrogen receptor
**ERK**	Extracellular signal-regulated kinase
**FBS**	Fetal bovine serum
**GAPDH**	Glyceraldehyde-3-phosphate dehydrogenase
**GDGFB**	Platelet derived growth factor B
**HER2**	Human epidermal growth factor receptor 2
**IHC**	Immunohistochemical
**ITGB3**	Integrin β3
**JNK**	Jun N-terminal kinase
**MAPK**	Mitogen-activated protein kinase
**MMPs**	Matrix metalloproteases
**MSCs**	Mesenchymal stem cells
**OPG**	Osteoprotegerin
**OS**	Overall survival
**PCR**	Polymerase chain reaction
**PPS**	Post-progression survival
**PR**	Progesterone receptor
**PTEN**	Phosphatase and tensin homolog deleted on chromosome ten
**PTHrP**	Parathyroid hormone-related protein
**PVDF**	Polyvinylidene fluoride
**qPCR**	Real-time quantitative PCR
**RANK**	Receptor activator of nuclear factor kappa B
**RANKL**	Receptor activator of nuclear factor kappa-B ligand
**RFS**	Relapse free survival
**RIPA**	Radio-immunoprecipitation assay
**RSEM**	RNA-Seq by Expectation-Maximization
**RUNX2**	Runt-Related Transcription Factor 2
**SDS-PAGE**	Sodium dodecyl sulfate-polyacrylamide gel electrophoresis
**TCGA-BRCA**	The Cancer Genome Atlas breast cancer cohort
**TMA**	Tissue microarray
**TNBC**	Triple negative breast cancer
**TPM**	Transcripts per million
**VEGF**	Vascular endothelial growth factor

## Data Availability

The TCGA-BRCA dataset can be found via the following link: https://portal.gdc.cancer.gov/projects/TCGA-BRCA.
